# Acute Seizures and Aphasia in a Patient With Suspected Systemic Lupus Erythematosus: A Diagnostic Puzzle Unveiling Diffuse Hemispheric Glioma, H3 G34-Mutant

**DOI:** 10.7759/cureus.111506

**Published:** 2026-06-25

**Authors:** Xiaojun Li, Ibrahim S Tuna, Marie Rivera-Zengotita, Hans Shuhaiber

**Affiliations:** 1 Department of Neurology, University of Florida, Gainesville, USA; 2 Department of Radiology, University of Florida, Gainesville, USA; 3 Department of Pathology, Immunology, and Laboratory Medicine, University of Florida, Gainesville, USA

**Keywords:** diffuse hemispheric glioma, encephalitis, h3-3a, histone, seizure

## Abstract

Diffuse hemispheric glioma, H3 G34-mutant (DHG-H3-G34), is a rare, newly defined WHO grade 4 glioma. Its nonspecific symptoms and radiologic heterogeneity can mimic infectious, autoimmune, and paraneoplastic neurologic disorders, complicating timely diagnosis. We present a case of a 20-year-old man with a history of suspected systemic lupus erythematosus who presented with acute encephalopathy, seizures, and aphasia. He underwent an extensive workup to evaluate for possible encephalitis. Biopsy ultimately revealed the diagnosis of DHG-H3-G34. This case highlights the importance of maintaining a broad differential in young patients with acute neurologic decline. Early consideration of neoplasms is crucial to avoid diagnostic delays in high-grade gliomas.

## Introduction

Diffuse hemispheric glioma, H3 G34-mutant (DHG-H3-G34), is an infiltrative high-grade (WHO grade 4) glioma included under the pediatric-type diffuse high-grade gliomas in the most recent edition of the WHO Classification of Central Nervous System (CNS) Tumors (2021, 5th edition) [1]. It is defined by missense mutations in codon 34 of the *H3-3A* (previously *H3F3A*) gene, resulting in a glycine-to-arginine (G34R) or glycine-to-valine (G34V) substitution of histone H3. These mutations are specific to this type of glioma. They are thought to alter chromatin regulation and disrupt normal gene expression and DNA repair, thereby contributing to the tumor's unique biology [2]. The tumor predominantly affects children, adolescents, and young adults, though ages ranging from 8 to 70 have been reported [3]. Common presentations include seizures, focal deficits, and acute intracranial hypertension [4]. On neuroimaging, the tumors are predominantly supratentorial, most often involving the frontal, temporal, or parietal lobes [3,5,6]. Only a small proportion of tumors show marked contrast enhancement. They also exhibit variable patterns of edema, necrosis, and hemorrhage. The nonspecific symptoms and variable radiologic features of DHG-H3-G34 can mimic inflammatory and infectious conditions, particularly in the presence of systemic conditions.

We report the case of a 20-year-old man with a history of suspected systemic lupus erythematosus (SLE) who presented with acute encephalopathy, aphasia, and seizure. An extensive workup was performed to differentiate encephalitis from CNS tumors. Ultimately, a biopsy was performed, which confirmed the diagnosis of DHG-H3-G34. This case underscores the capacity of DHG-H3-G34 to mimic various CNS disorders and emphasizes the importance of maintaining a broad differential diagnosis in young patients presenting with acute neurologic deterioration.

## Case presentation

A 20-year-old man with a past medical history of suspected SLE and suspected lupus nephritis initially presented to an outside hospital with a sudden-onset severe throbbing headache in the left temporo-occipital area with nausea and emesis. He was also noted to have auditory hallucinations, confusion regarding his location, an inability to recognize his parents, incoherent speech, and an inability to comprehend reading. Upon presentation, he had a witnessed tonic-clonic seizure lasting a few minutes. Magnetic resonance imaging (MRI) showed asymmetric gyriform and subcortical edema, with cortical expansion in the left posterior temporal lobe and subtle patchy enhancement (Figure 1). Electroencephalography (EEG) was reportedly unremarkable.

**Figure 1:**
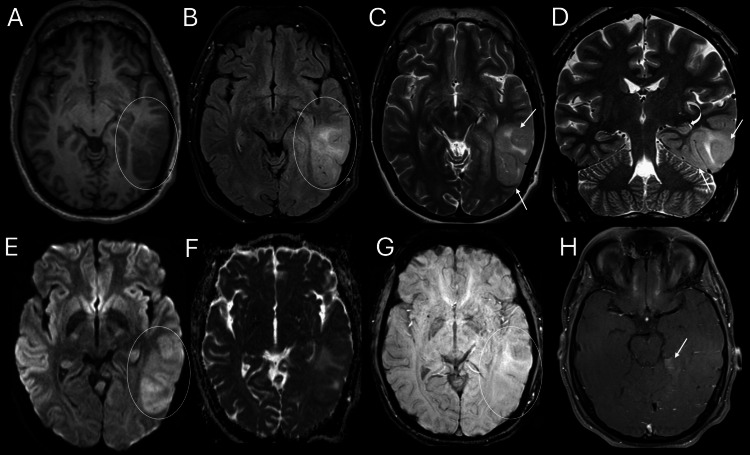
Radiological findings Axial precontrast 3D T1 MPRAGE (A), axial T2 FLAIR (B), axial T2-weighted (C), coronal T2-weighted (D), axial DWI (E) and corresponding ADC map (F), axial SWI (G), and axial postcontrast T1 fat-saturated image (H) are shown. Images demonstrate an asymmetric, predominantly gyriform abnormality in the left posterior temporal lobe (oval circles and arrows), measuring approximately 60 × 31 mm. The lesion appears hypointense on T1 (A), hyperintense on T2 and FLAIR (B-D), and is associated with cortical and subcortical expansion and surrounding edema. There is no significant restricted diffusion (E), nor evidence of hemorrhage on SWI (G). Postcontrast imaging shows mild, patchy enhancement in the medial portion of the lesion (H). Additionally, the left hippocampus shows mild edema and blurred internal architecture (curved arrow in D), suggestive of recent seizure activity. MPRAGE: magnetization-prepared rapid gradient-echo, FLAIR: fluid-attenuated inversion recovery, DWI: diffusion-weighted imaging, ADC: apparent diffusion coefficient, SWI: susceptibility-weighted imaging

Previously, the patient was suspected of having SLE based on a positive antinuclear antibody (ANA, 1:160, nuclear speckled), a weakly positive dsDNA antibody, and a limited kidney biopsy showing focal crescentic and proliferative glomerulonephritis suggestive of class III + V lupus nephritis. However, the patient lacks other typical symptoms of SLE, and repeat serology was negative, making the diagnosis of SLE questionable. On presentation, he was taking mycophenolate mofetil and hydroxychloroquine.

Given the diagnosis of suspected SLE, use of immunosuppressants, and cortical involvement with enhancement, diagnoses including neuropsychiatric SLE, infectious encephalitis, and infiltrative tumor were considered. He underwent lumbar puncture. Cerebrospinal fluid (CSF) studies demonstrated normal white blood cell count, protein, and glucose; negative oligoclonal bands; negative angiotensin-converting enzyme; and a negative infectious workup. Cytology, flow cytometry, and autoimmune and paraneoplastic testing were not performed at that time. Levetiracetam was started. After returning to his mental baseline, he was discharged with a plan for neurology follow-up.

The next day, his symptoms recurred, and he was transferred to our hospital. MRI demonstrated findings that were stable compared with the previous study. EEG captured left temporal electrographic seizures. Levetiracetam was increased, and a short course of empiric acyclovir was started due to concern for herpes simplex virus (HSV) encephalitis. Repeat CSF studies showed normal white blood cells, protein, and glucose with negative cytology and flow cytometry. Infectious workup, including HSV, as well as autoimmune and paraneoplastic workup, was all negative. Given the concern for an infiltrative tumor on MRI, the patient underwent a left craniotomy for a brain biopsy. Intraoperative pathology favored neoplasm. He subsequently underwent subtotal resection of the tumor. Pathology revealed an infiltrating glioma with increased mitotic activity and focal calcifications (Figure 2). No definitive microvascular proliferation or necrosis was identified. By immunohistochemistry, the tumor was negative for *IDH1* (R132H) and *ATRX* mutations.

**Figure 2:**
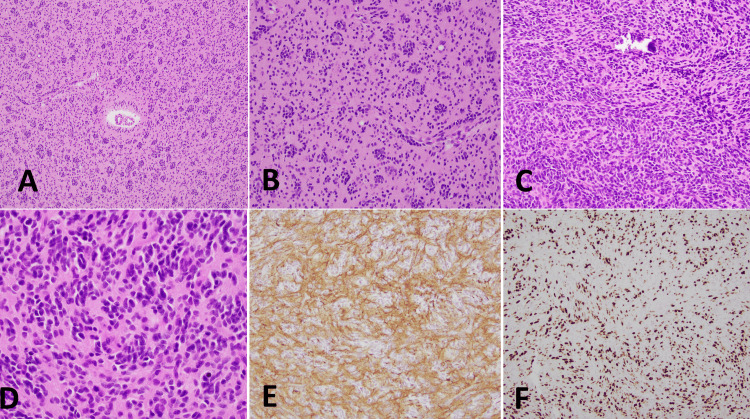
Histopathological findings H&E-stained sections revealed a hypercellular diffuse glioma (A, ×100) demonstrating extensive perineuronal satellitosis and perivascular tumor spread (B, ×200). Markedly cellular areas showed tumor cells with a high nuclear-to-cytoplasmic ratio, nuclear molding, and calcifications (C, ×200), apoptosis, and increased mitotic activity (D, ×400). Tumor cells and their processes were immunoreactive for glial fibrillary acidic protein (E, ×200) and showed increased Ki-67 proliferation index (F, ×200). H&E: hematoxylin and eosin

Next-generation sequencing identified mutations in *H3F3A* (G34R), the *TERT* promoter, and *PDGFRA*, as well as loss of heterozygosity at 17q. There were also subclonal mutations in *EGFR* and *BCOR*. No *IDH1/IDH2* mutations were detected. A diagnosis of diffuse hemispheric glioma, H3 G34-mutant (DHG-H3-G34), CNS WHO grade 4, was rendered. Postoperatively, the patient had breakthrough seizures, requiring increased levetiracetam, as well as the addition of lacosamide and valproic acid. He received six weeks of concurrent proton therapy and temozolomide, followed by temozolomide and briefly tumor-treating fields. Repeat MRIs showed no evidence of progressive disease as of nine months following resection. He is undergoing further evaluation for matched molecular therapy.

## Discussion

The variable and nonspecific presentation of DHG-H3-G34 can mimic a broad range of neurologic conditions, posing significant diagnostic challenges. In this case, the presentation of acute encephalopathy, aphasia, and seizures, along with temporal edema without a clearly delineated mass, can be explained by a broad differential diagnosis, including structural, infectious, autoimmune, and paraneoplastic etiologies. CSF studies, while diagnostically important, were essentially negative and did not provide additional diagnostic clues. This was further confounded by the patient’s history of suspected SLE, as CNS involvement of SLE can present similarly. Common symptoms include encephalopathy, hallucinations, seizures, stroke, and headache. MRI findings are highly variable. In lupus angiitis, MRI may show hypointensities on T1-weighted sequences, hyperintensities on T2-weighted sequences, and variable enhancement. While evaluation for possible encephalitis is warranted in this case, the negative infectious, autoimmune, and paraneoplastic studies should raise suspicion for an alternative etiology. Prompt neurosurgery referral for tissue diagnosis should be considered. Ultimately, definitive diagnosis requires histopathologic and molecular analyses.

Radiographically, on MRI, DHG-H3-G34 characteristically presents as a large supratentorial hemispheric mass with T2/FLAIR hyperintensity, restricted diffusion, absent or only faint gadolinium enhancement (up to 79%), and variable intratumoral hemorrhage (30.0-42.2%) [6,7]. These features frequently mimic lower-grade gliomas, making the condition susceptible to misdiagnosis and diagnostic delay. Advanced imaging with ^18^F-FET PET and MR spectroscopy may provide additional clues to high-grade biology [6-8], but these modalities are not routinely obtained in the initial workup. While significant restricted diffusion, hemorrhage, or ring enhancement are hallmarks that typically raise suspicion for a WHO grade 4 glioma, the absence of these features in our case made a specific radiographic diagnosis exceedingly difficult. Ultimately, a tissue biopsy was essential to establish the diagnosis, underscoring that DHG-H3-G34 remains a histomolecular diagnosis that cannot be reliably made on imaging alone.

Histologically, DHG-H3-G34 typically shows a glioblastoma-like pattern, characterized by a hypercellular, infiltrating astrocytic neoplasm with high mitotic activity, microvascular proliferation, and/or necrosis. In some cases, DHG-H3-G34 resembles CNS embryonal tumors, consisting of monotonous tumor cells with a high nuclear-to-cytoplasmic ratio [3,9]. Concurrent alterations in oncogenes and tumor suppressors are common, with mutations in *TP53*, *ATRX*, and *PDGFRA* being the most common [3,9]. Mutations in the *TERT* promoter gene, which were seen in this case, have also been reported in a small subset of patients [3,10]. While previous *EGFR* alterations were identified as amplifications [3,4,10], our patient exhibits a missense mutation at c.664C>T (p.R222C), further adding to the heterogeneity of the tumor’s genomic landscape.

There is currently no standardized treatment for DHG-H3-G34. The typical approach involves maximal safe surgical resection followed by adjuvant radiotherapy and chemotherapy (most commonly temozolomide). Targeted therapies are currently under investigation. The overall prognosis remains poor, with a median overall survival of approximately 21 months [3,11]. Positive prognostic factors identified include gross total resection and *MGMT* promoter methylation status. Poorer outcomes have been associated with the G34V mutation (rather than G34R), *PDGFRA* amplification or mutation, and *EGFR* amplification [3,5,11].

Overall, this case emphasizes the need for early consideration of neoplasm in patients with unexplained focal neurological deficits and seizures, even in the presence of an autoimmune background. This case also highlights the current diagnostic limitations of neuroimaging and CSF studies in DHG-H3-G34. Although DHG-H3-G34 is a recently described glioma, increased awareness among physicians is essential for prompt diagnosis and initiation of treatment.

## Conclusions

DHG-H3-G34 is a recently described high-grade glioma that presents with nonspecific symptoms and variable radiologic and histopathologic features, posing diagnostic challenges and delaying treatment. Early consideration of a neoplasm and prompt biopsy are warranted when imaging and CSF findings are inconclusive. Further research is needed to refine diagnostic accuracy and optimize treatment strategies.
